# High Efficacy of Stem Cell Mobilization With Etoposide+Cytarabine Plus G-CSF in Patients With Multiple Myeloma

**DOI:** 10.3389/fonc.2022.825550

**Published:** 2022-01-27

**Authors:** Zhijuan Zhu, Xiaofan Li, Yiping Liu, Ping Chen, Xianling Chen, Hua Li, Jiafu Huang, Yuanzhong Chen, Nainong Li

**Affiliations:** ^1^ Fujian Institute of Hematology, Fujian Provincial Key Laboratory on Hematology, Fujian Medical University Union Hospital, Fuzhou, China; ^2^ Translational Medicine Center on Hematology, Fujian Medical University, Fuzhou, China

**Keywords:** Etoposide, Cytarabine, granulocyte colony-stimulating factor, stem cell mobilization, multiple myeloma

## Abstract

**Background:**

Efficient mobilization of CD34+ hematopoietic stem cells plays a vital role in successful autologous stem cell transplantation (ASCT) in patients with multiple myeloma (MM), especially in cases with high-risk cytogenetic recommended for tandem ASCT. However, the optimal mobilization strategy remains a matter of debate in the era of lenalidomide. The combination of etoposide with Cytarabine plus G-CSF as a novel mobilization regimen in MM has not been reported previously.

**Methods:**

This research retrospectively studied mobilization efficacy and safety using etoposide combined with Cytarabine (etoposide 50–100 mg/m^2^, qd d1–3; AraC 0.5 g/m^2^, q12h d1~3) plus G-CSF (5 µg/kg/day, from d5 until the day of apheresis) in 128 patients with MM. 70(54.7%) patients received lenalidomide-based induction regimens treatment

**Results:**

A median of 27.75×10^6^ CD34+ cells/kg was collected in the first apheresis, and 28.23×10^6^ CD34+ cells/kg were collected overall. Of the 128 patients, all achieved adequate collection (≥2×10^6^ CD34+ cells/kg), 121(94.5%) achieved optimal collection for single ASCT (≥5×10^6^ CD34+ cells/kg), and 114(89.1%) harvested optimal collection for tandem ASCT (≥10×10^6^ CD34+ cells/kg). In particular, the target yield of optimal collection for tandem ASCT was reached in 82.8% (106/128) by a single apheresis procedure. 14 patients obtained deeper response post mobilization. In multivariate analysis, cycles of prior chemotherapy independently affected the optimal achievement of CD34+ cells (*p*=0.004, OR 0.695, 95% CI 0.544~0.888). Previous lenalidomide exposure did not significantly impair CD34+ cells collection. Although 68% episodes of antibiotic usage were observed, no severe infection or treatment-related mortality occurred.

**Conclusion:**

Stem cell mobilization with Etoposide + Cytarabine plus G-CSF was highly efficient and safe in patients with MM, which could be considered in high-risk MM patients who were referred for tandem ASCT.

## Introduction

High-dose chemotherapy followed by autologous stem cell transplantation (ASCT) is an important option for transplant-eligible patients with multiple myeloma (MM) (1, 2), which can significantly improve patients’ outcomes. Moreover, tandem ASCT is currently recommended in patients with MM and high-risk disease according to the presence of cytogenetic abnormalities (3). Adequate collection of CD34+ hematopoietic stem cells (HSCs) is important for successful engraftment post-ASCT, and high collection is associated with faster hematologic recovery, reduced infection risk, and improved overall survival (4, 5).

At present, the commonly utilized mobilization strategies include cytokine mobilization such as granulocyte colony-stimulating factor (G-CSF); chemo-mobilization using chemotherapy, followed by cytokine administration, such as the use of cyclophosphamide (Cy) prior to G-CSF; and CXCR4 inhibitor plerixafor mobilization (6). Cy plus G-CSF is currently a wildly used regimen for HSC mobilization in patients with MM. Although published literatures on these mobilization approaches have reported encouraging mobilization efficacy, approximately 5%–40% of patients fail to collect adequate dose of HSCs (7, 8) because of tumor burden, previous treatments such as lenalidomide, and age of patients (5, 6). Furthermore, the use of Cy during chemo-mobilization has been linked to increased morbidity such as febrile neutropenia (9), and plerixafor administration is associated with high cost, which has led to its restricted use as a pre-emptive regimen for patients at high risk for mobilization failure (10, 11). Thus, the optimal regimen for the cost-efficient collection of HSCs remains a challenge, particularly in patients with MM who were referred for tandem ASCT.

Etoposide or Cytarabine (AraC) plus G-CSF administrated as chemo-mobilization had been reported in patients with MM for the past years with potential outcomes (12–16). At present, etoposide (375 mg/m^2^) plus G-CSF was reported as an effective and safe method of mobilization in patients with MM (14). The median number of collected CD34+ cells was 5.58×10^6^/kg, and the adequate harvest rate was 96.9%, which was higher than that of HD-Cy. However, only 71.9% of patients achieved the optimal harvest. Moreover, AraC plus G-CSF was more efficient than Cy + G-CSF as a stem cell mobilization regimen of MM (15). Compared with Cy, intermediate-dose AraC as a mobilization regimen achieved a higher peak concentration of CD34+ cells in the blood and higher CD34+ yield. Thus, we administered etoposide combined with AraC (EA) plus G-CSF clinically for patients with MM who were eligible for ASCT, to improve the cost-efficacy and safety of HSC mobilization. In this study, we aimed to retrospectively analyze the efficacy and safety of our novel protocol and explore factors affecting mobilization outcomes.

## Patients and Methods

### Patients

We retrospectively analyzed 128 patients with newly diagnosed MM who underwent peripheral blood stem cell (PBSC) mobilization for ASCT at Fujian Medical University Affiliated Union Hospital between March 2013 and October 2021. Mobilization for stem cell collection was conducted in patients eligible for ASCT. Patients undergoing mobilization for tandem ASCT or ASCT at the outset of salvage therapy for relapsed disease were also included. The treatment response was determined using the International Myeloma Working Group criteria (17). Cytogenetic risk was classified according to Mayo Stratification for Myeloma and Risk-Adapted Therapy 3.0 (mSMART3.0). Adverse events were graded using The Common Terminology Criteria for Adverse Events Version 4.0 (CTCAEv4). Deep response was defined as very good partial remission (VGPR) or better. The study protocol was approved by the ethics committee of Union Hospital, Fujian Medical University. Informed consent was obtained from all patients before the study.

### Mobilization Regimens and Leukapheresis

All patients received EA (etoposide 50–100 mg/m^2^, qd d1–3; AraC 0.5 g/m^2^, q12h d1~3), and G-CSF (5 µg/kg/day) was administered from d5 until the day of apheresis. Etoposide dosage was adjusted according to renal function. Collection was initiated according to the value of CD34+ cells or hematopoietic progenitor cell count in the peripheral blood, and the primary yield target was ≥2.0×10^6^/kg for single ASCT and ≥4×10^6^/kg for tandem ASCT. During 2013~2016, the first 15 patients stayed in the hematology ward during the whole mobilization period. Since 2017, the subsequent 113 patients were hospitalized for 3 days for chemotherapy administration, and then they received G-CSF daily administered for 6 days in outpatient clinic. All patients were asked to re-hospitalize on d10 and discharged after apheresis finished and meet the discharge standard. All patients received peripherally inserted central catheter (PICC) for the administration of mobilization chemotherapy, as well as for the subsequent transplantation condition regimen.

### ASCT and Engraftment

High-dose melphalan (140 mg/m2 IV) was used as a conditioning regimen for patients with MM. G-CSF (5 µg/kg/day) was administrated 5 days post-ASCT and continued until neutrophil was engrafted. Neutrophil and platelet engraftment was defined according to the guideline on post-transplant essential data from the Center for International Blood and Marrow Transplantation Research (CIBMTR).

### Efficacy Assessment

Optimal mobilization was defined as a mobilized CD34+ cell count of ≥5×10^6^/kg, and adequate mobilization was defined as a mobilized CD34+ cell count of ≥2×10^6^/kg (6, 18). Adverse events included grade 4 hematologic toxicity (according to the Common Terminology Criteria for Adverse Events version 4.0), and infection complication was defined as the presence of antibiotic administration because of infection. Importantly, no antibiotics were used as prophylaxis.

### Statistical Analysis

Data analysis was performed using the IBM Statistical Package for Social Sciences version 25.0. Normality of data was tested using the kolmogorov-smirnov Z test. Categorical variables were compared by the χ2 test or exact test. The chi-square test or the Mann-Whitney U test was used to compare differences of variables between groups. To identify predictive factors for optimal harvest, univariate, and multivariate analyses by logistic regression were performed. A two-sided value of *p*<0.05 was considered statistically significant for all analyses.

## Results

### Patient Characteristics

A total of 128 patients were enrolled in this study. The median age was 56 years (range: 26–67 years), and 23.4% (30/128) patients were aged over 60 years. 13.3% (17/128) of patients weighed over 75 kg. Before mobilization, 99 (77.3%) patients achieved deep response, 42 (32.8%) patients received ≥2 lines of preceding therapy and 70 (54.7%) received lenalidomide-based induction regimens treatment. 64.3% patients who received ≥2 lines of preceding therapy achieved deep response (VGPR or better), lower than that of patients received first line of treatment (83.7%, *p*=0.014). Moreover, the median cycle of prior chemotherapies was 4. Totally, 9 patients were diagnosed with chronic kidney disease (1 in CKD2 stage, 4 in CKD3 stage and 4 in CKD4 stage) prior mobilization chemotherapy administration. Patients on dialysis were excluded for using EA regimen and mobilization. Patient characteristics are summarized in **Table 1**.

**Table 1:** Patient characteristics.

Variables	Total
	(*n*=128)
Age, years, median(range)	56(26~67)
Sex, male, *n*(%)	79(61.7%)
Weight >75kg, *n*(%)	17(13.3%)
ISS stage, *n*(%)	
1	36(28.3%)
2	46(36.2%)
3	45(35.4%)
Diagnosis, *n*(%)	
IgG	69(53.9%)
IgA	28(21.9%)
Light chains	25(19.5%)
IgD	3(2.3%)
IgM	2(1.6%)
biclonal gammopathy^a^	1(0.8%)
Cytogenetic risk^b^, *n*(%)	
high risk	58(45.3%)
standard risk	49(38.3%)
NA	21(16.4%)
Month from diagnosis to mobilization, median (range)	4(2~74)
Lenalidomide-included therapy, *n*(%)	70(54.7%)
Lines of preceding therapy, *n*(%)	
1	86(67.2%)
2	33(25.8%)
3	8(6.3%)
4	1(0.8%)
Cycles of preceding therapy, median(range)	4(2~16)
Response to induction treatment, *n*(%)	
VGPR or better	99(77.3%)
PR	22(17.2%)
MR	2(1.6%)
SD	3(2.3%)
PD	2(1.6%)

MM, multiple myeloma; ISS, international staging system; VGPR, very good partial remission. ^a^, IgG and IgA biclonal gammopathy. ^b^, risk classification according to mSMART3.0; PR, partial remission; MR, minimal remission; SD, stable disease; PD, progressive disease.

### Efficacy of Mobilization

CD34+ cells were detected in all PBSCs collected from the patients using flow cytometry. The median peak number of circulating CD34+ cells was 80/ul, and the median number of overall collected CD34+ cells was 28.23×10^6^/kg. There was no difference in overall collected CD34+ cells between patients who were exposed to previous lenalidomide and those were not (25.59×10^6^/kg and 33.27×10^6^/kg, *p*=0.34). Of the patients, all achieved adequate collection, and 94.5% (121/128) achieved optimal collection. Moreover, 89.1% (114/128) of patients obtained at least 10×10^6^/kg CD34+ cells, thereby achieving optimal mobilization target for tandem ASCT. In particular, 124(96.9%) patients with MM yielded adequate harvest, and 116(90.6%) patients achieved optimal mobilization through the first apheresis session. A single apheresis was sufficient to collect at least 10×10^6^/kg CD34+ cells in 82.8% (106/128) of patients with MM. Furthermore, the overall median interval of mobilization to collection was 14 days. The median length of hospitalization was 15 days (range: 13~18 days) for the first 15 patients during 2013~2016, and 8 days (range: 5~23 days) for the subsequent 112 patients during 2017~2021. The results are summarized in **Table 2**.

**Table 2:** Mobilization efficacy.

Variables	Total	Lenalidomide explosure(*n*=70)
	(*n*=128)	
Total CD34+ cells collected, ×10^6^/kg, median (range)	28.23(2.51~464.3)	25.59(2.57~118.77)
Total Collection target, *n* (%)		
≥2×10^6^ CD34+ cells/kg	128(100%)	70(100%)
≥5×10^6^ CD34+ cells/kg	121(94.5%)	66(94.3%)
≥10×10^6^CD34+ cells/kg	114(89.1%)	65(92.9%)
Day 1 pheresis day target, *n* (%)		
≥2×10^6^ CD34+ cells/kg	124(96.9%)	67(95.7%)
≥5×10^6^ CD34+ cells/kg	116(90.6%)	62(88.6%)
≥10×10^6^ CD34+ cells/kg	106(82.8%)	60(85.7%)
Day 1and 2 pheresis target, *n* (%)		
≥2×10^6^ CD34+ cells/kg	127(99.2%)	70(100%)
≥5×10^6^ CD34+ cells/kg	121(94.5%)	66(94.3%)
≥10×10^6^ CD34+ cells/kg	114(89.1%)	65(92.9%)
Days of leukapheresis initiation, median(range)	14(8~21)	14(8~21)
Days of pheresis, *n* (%)		
1 day	107(83.6%)	58(82.9%)
2 days	20(15.6%)	12(17.1%
3 days	1(0.8%)	0(0%)

MM, multiple myeloma.

Age, ISS stage, prior lenalidomide exposure, deep response to induction, the number of peripheral blood CD34+ cells at the first day of apheresis, cycles of prior therapy were considered for multivariate analysis to explore the factors associated with failure to achieve optimal mobilization. The result showed that cycles of prior chemotherapy independently affected the optimal achievement of CD34+ cells in patients with MM (*p*=0.004, OR 0.695, 95% CI 0.544~0.888). Previous lenalidomide exposure did not significantly decrease the absolute number of CD34+cells collected.

### Adverse Events

The most common complication was hematologic toxicity. The mean duration of grade 4 neutropenia was longer compared to that of grade 4 thrombocytopenia (3.36 days and 3.00 days, respectively, *p*<0.01). However, no bleeding episode was observed. A relatively shorter duration of thrombocytopenia indicated fewer amounts of platelet transfusion (median1.6, range: 0~6) before apheresis. During mobilization, 87 (68%) patients received antibiotic administration, no mortality occurred during mobilization. Generally, 85 patients suffered grade 2~3 infections, while grade 4 infections were found in only 2 patients (septic shock complication). The infections sites include respiratory tract, oral cavity mucosa, septicemia, soft tissue infection, digestive tract and febrile neutropenia unknown origin. The common infections were respiratory tract and fever unknown origin. And the median duration of antibiotic administration was 4 days (range 1~13 days). Toxicity information are summarized in **Table 3**.

**Table 3:** Common toxicities.

Variables	Total
	(*n*=128)
Days of Grade 4 Neutropenia, median (range)	3(0~6)
Days of Grade 4 Thrombocytopenia, median (range)	3(0~6)
Platelet transfusions, median (range)	1.6(0~6)
Erythrocyte transfusions,median (range)	0(0~4)
Infection, *n* (%)	
grade 2	4(3.1%)
grade 3	81(63.3%)
grade 4	2(1.6%)

MM, multiple myeloma.

### Hematologic Recovery After ASCT

A total of 125 patients underwent ASCT as planned, except for 3 patients who are still waiting in line for receiving ASCT. In particular, 70 MM patients underwent tandem ASCT as planned. All patients achieved hematologic recovery post-HSC infusion, and both the median interval time of neutrophil and platelet engraftment was 11 days. Importantly, we found 14 patients achieved deeper responses after mobilization. Detailed data concerning hematologic reconstitution are summarized in **Table 4**.

**Table 4:** Hematologic recovery after ASCT.

Variables	Total
	(*n*=125)
Months from diagnosis to transplantation,	6(4~75)
median(range)	
Day of reach, median (range)	
Neutrophils ≥0.5×10^9^/ul	11(0~38)
PLT ≥20×10^9^/ul	11(0~48)
Platelet transfusions, median (range)	1.8(0~20.6)
Erythrocyte transfusions, median (range)	0(0~14.5)

## Discussion

The recommended adequate mobilization is at least 2×10^6^ CD34+ cells/kg, as an infusion less than this value can impair engraftment (6). Moreover, this target yield must be higher in patients with MM because of a possible second transplantation, such as tandem ASCTs in high-risk diseases (3). Successful mobilization of PBSCs predicts favorable outcomes in patients with MM (4, 6), whereas poor PBSCs mobilization is significantly associated with shortened progression free survival and overall survival compared with good mobilizers (5, 19). Hence, the recommended optimal CD34+ cell dose is at least 5×10^6^/kg (8, 20). Using modern agents such as lenalidomide’s induction as first-line treatment in transplant-eligible patients, impaired mobilization after its administration has been raised (7, 21–23). However, stem cell collection through repeated apheresis may physically and economically burden patients and increase complications. Thus, the goal of mobilization is to cost-effectively collect sufficient HSCs using the least number of apheresis procedures with minimal mobilization-related complications.

Various attempts have been proposed to increase the efficacy of mobilization regimens. One option is the administration of an additional chemotherapy regimen plus G-CSF. Among chemo-mobilization protocols, intermediate-dose CY has been widely used (9, 24). Recently, single-dose etoposide or intermediate-dose AraC as mobilization regimens with G-CSF has been reported to reach encouraging results compared with CY in patients with MM and lymphoma (13–15, 25). Our findings showed that the use of etoposide combined with AraC plus G-CSF regimen could increase the adequate and optimal collection rates in patients with MM (100% vs. 94.5%) by 1.2 and 1.1 apheresis procedures (mean), respectively. Importantly, 89.1% of patients with MM reached optimal mobilization for tandem ASCT (≥10×106 CD34+ cells/kg) through minimal apheresis. This advantage contributed to the reduction of stem cell mobilization costs and apheresis-associated complications.

Risk factors for mobilization failure, including aging, prior treatment with lenalidomide, heavily preceding treatment, and diagnosis have been established (6, 19, 26). In our study, we considered cycles of prior chemotherapy as an independent risk factor influencing the achievement of optimal harvest in patients with MM, which implied that the novel regimen might overcome the negative impact of lenalidomide on mobilization. This result was consistent with recent reports (27). Commonly used mobilizations based on chemotherapy have been observed with 0%–73% of grade 4 neutropenia and 0%–50% of grade 4 thrombocytopenia (12, 13, 15). Although the duration of grade 4 hematological toxicity or antibiotic administration was short, and no mobilization-related mortality was observed, the occurrences were more common in our protocol. As intermediate-dose AraC (1.6 g/m^2^) was associated with a high adequate mobilization rate with slight hematologic complication (12, 13, 15), decreasing the AraC’s dosage in our regimen might be an option to reduce complications without affecting mobilization efficacy.

Whether chemotherapy contributes to the outcome of ASCT and the depth of response has been discussed over years because it is originally intended to reduce tumor burden by additional exposure to cytotoxic agents. However, a previous retrospective analysis of 968 patients with MM from the CIBMTR database reported similar prognosis of 3-year progression-free survival (43% vs. 40%, *p*=0.33) and overall survival (82% vs. 80%, *p*=0.43) between G-mobilization and chemo-mobilization (28). Another work demonstrated that chemotherapy-based stem cell mobilization does not contribute to disease control in the era of novel induction regimens (29, 30). In our study, 117 patients with MM were assessed disease status before mobilization and prior autologous transplantation, respectively. We observed that 14 patients obtained deeper response depth post mobilization, which indicated that our new protocol might contribute to disease control. However, it needs further prospective clinical investigation to confirm the current result. High-dose cytotoxic agents as part of mobilization might damage human mesenchymal stem cells, resulting in delayed engraftment after ASCT. In our study, the median days to neutrophil and platelet engraftment post-transplantation were comparable with those recently published (12, 14, 15, 28).

## Conclusions

Collectively, we have demonstrated that etoposide combined with AraC plus G-CSF is an efficient and safe regimen for CD34+ PBSC mobilization in patients with MM. This novel regimen might be an option particularly for high-risk MM patients who are referred for tandem ASCTs and cases where use plerixafor in MM is difficult. Nevertheless, this research is not a prospective randomized study that directly compares the cost-efficacy and safety of etoposide + AraC plus G-CSF with common mobilization protocols. Therefore, our conclusion must be confirmed prospectively with a larger randomized trial in the future.

## Data Availability Statement

The original contributions presented in the study are included in the article/supplementary material. Further inquiries can be directed to the corresponding authors.

## Ethics Statement

The studies involving human participants were reviewed and approved by the ethics committee of Union Hospital, Fujian Medical University. The patients/participants provided their written informed consent to participate in this study.

## Author Contributions

ZZ, XL: data collection, data analysis and interpretation, manuscript writing, manuscript revision, final approval of manuscript. YL: data collection, data analysis and interpretation, final approval of manuscript. PC, XC, HL, JH: data analysis and interpretation, final approval of manuscript. NL, YC: study design, data analysis and interpretation, manuscript revision, final approval of manuscript. All the authors read and approved the final manuscript.

## Funding

This work was supported by Department of Science and Technology of Fujian Province Project (2017I0004 and 2017Y9057); Fujian provincial health technology project (2021ZD01005); Top Notch Innovative Talents Project and Fujian Project(2016Y9025 and 2016J06018); National Key Clinical Specialty Discipline Construction Program(2021-76) ; This work was also sponsored by Fujian Provincial Clinical Research Center for Hematological Malignancies (2020Y2006); and Fujian Provincial Key Clinical Specialty Discipline Construction Program, P. R.C.

## Conflict of Interest

The authors declare that the research was conducted in the absence of any commercial or financial relationships that could be construed as a potential conflict of interest.

## Publisher’s Note

All claims expressed in this article are solely those of the authors and do not necessarily represent those of their affiliated organizations, or those of the publisher, the editors and the reviewers. Any product that may be evaluated in this article, or claim that may be made by its manufacturer, is not guaranteed or endorsed by the publisher.
